# Carbohydrate-Active Enzymes of a Novel Halotolerant *Alkalihalobacillus* Species for Hydrolysis of Starch and Other Algal Polysaccharides

**DOI:** 10.1128/spectrum.01078-22

**Published:** 2022-07-11

**Authors:** Matan Masasa, Ariel Kushmaro, Helena Chernova, Nadav Shashar, Lior Guttman

**Affiliations:** a Marine Biology and Biotechnology Program, Department of Life Sciences, Ben-Gurion University of the Negev Eilat Campus, Eilat, Israel; b Israel Oceanographic and Limnological Research, The National Center for Mariculture, Eilat, Israel; c Avram and Stella Goldstein-Goren Department of Biotechnology Engineering, Ben-Gurion University of the Negev, Beer-Sheva, Israel; d The Ilse Katz Center for Nanoscale Science and Technology, Ben-Gurion University of the Negev, Beer Sheva, Israel; e School of Sustainability and Climate Change, Ben-Gurion University of the Negev, Beer-Sheva, Israel; Penn State University

**Keywords:** *Alkalihalobacillus* sp., halotolerant bacterium, starch hydrolysis, carbohydrate-active enzymes, α-amylase, algal polysaccharides

## Abstract

Halotolerant bacteria capable of starch hydrolysis by their amylases will benefit various industries, specifically since the hydrolytic activity of current industrial amylases is inhibited or even absent in salt-rich or alkaline environments. Seeking novel enzymes, we analyzed the entire genome content of a marine bacterium isolated from the gut of sea urchins to compare it against other bacterial genomes. Conditions underlying α-amylase activity were examined *in vitro* at various salinities (0 to 4%) and temperatures (25°C to 37°C). Genomic analyses revealed the isolated bacterium as a new species of *Alkalihalobacillus*. Comparative analysis of the contents of carbohydrate-active enzymes revealed various α-amylases, each with its respective carbohydrate-binding module for starch hydrolysis. Functional analysis identified the hydrolysis of starch and the maltooligosaccharides maltose and dextrin into d- and UDP-glucose. The fastest growth and α-amylase production occurred at 3% salinity at a temperature of 30°C. The *Alkalihalobacillus* sp. consists of exclusive contents of α-amylases and other enzymes that may be valuable in the hydrolysis of the algal polysaccharides cellulose and laminarin.

**IMPORTANCE** Toward the discovery of novel carbohydrate-active enzymes that may be useful in the hydrolysis of starch, we examined a halotolerant bacterial isolate of *Alkalihalobacillus* sp. regarding its genomic content and conditions underlying the production of active α-amylases. The production of α-amylases was measured in bacterial cultures at relatively high temperature (37°C) and salinity (4%). The *Alkalihalobacillus* sp. revealed an exclusive content of amylases and other carbohydrate-active enzymes compared to other relevant bacteria. These enzymes may be valuable for the hydrolysis of algal polysaccharides. The enzymatic cascade of the *Alkalihalobacillus* sp. for starch metabolism allows polysaccharide degradation into monosugars while preventing the accumulation of intermediate inhibitors of maltose or dextrin.

## INTRODUCTION

Starch is a polysaccharide composed of amylose and amylopectin and is the primary carbohydrate in cells of various organisms (1). Commercially speaking, raw starch that can be hydrolyzed to monosaccharides by amylases and glucoamylases is renewable and inexpensive (2). These enzymes consist of many subfamilies with diverse functional specifications such as temperature, pH, salinity, and substrate properties (3) to be considered in developing cost-effective commercial processes for starch hydrolysis (4). Industrial effluents from food, textile, and paper processing contain diverse starch types and high contents of soluble salts, which limit the activity or efficiency of many of the currently available amylases (2). In recent years, starch-rich seaweed like Gracilaria conferta, Laminaria digitate, or Ulva rigida gained significant attention concerning their use in biorefinery processes for feeds, cosmetics, and bioethanol (5). Amylases from novel halotolerant bacteria may improve the yields of valuable products from algal biorefinery processes and increase the commercial value of many algae (6). For example, cyclodextrin is a valuable product from starch hydrolysis as the medical industry widely uses it as a cholesterol-chelating agent (7). Another application of this oligosaccharide is its integration into biodegradable food packages, which helps to reduce the oxidation of volatile molecules (8). A recent estimation by Global Market Insight (https://www.gminsights.com/industry-analysis/global-cyclodextrin-market) proposed a global market value of cyclodextrin in 2020 of over $260 million. Improving the yields of such valuable products from algal biorefinery is expected to aid in the expansion of algal aquaculture (6).

Amylases (α- or β-amylases) are all glycoside hydrolases (GHs) that can break down starch polymers into oligo- and monosaccharides. The GH13 family consists of only α-amylases from 44 subfamilies of hydrolases, transglycosidases, and isomerases (3). Although they may vary in the type of substrate from amylose to amylopectin, all GH13 α-amylases share the three-dimensional (3-D) structure of the catalytic domain of the α-glucosidic linkage in the polymolecule (4). In many cases, GH13 α-amylases contain carbohydrate-binding modules (CBMs), which are required for the binding of the substrate (e.g., CBM20, -21, -25, -26, -34, -41, -45, -48, -53, and -58) (3).

Currently, bacteria are the primary source of industrial α-amylases (9) due to their variance in the starch substrate. While several members of the genus *Bacillus* produce commercial thermostable α-amylases (10), halophiles such as Pseudoalteromonas neustonica or Kocuria varians seem better candidates for halostable/halotolerant α-amylases (11, 12). α-Amylases of halotolerant bacteria have gained much less attention. Still, most are thermostable and produce oligosaccharides in low-water or nonaquatic solvents where hydrolytic reactions are inhibited (13). Therefore, searching for novel halostable bacterial amylases and other carbohydrate-active enzymes (CAZymes) is still relevant and was the primary aim of the current study. We hypothesized that the gut microbiome of algivorous sea urchins fed starch-rich algae contains cryptic bacteria capable of starch hydrolysis in salt-rich environments. We further hypothesized that such bacteria, if found, would also contain a pool of other important carbohydrate-active enzymes for the degradation of various polysaccharides that may be useful in biorefineries of marine algae.

## RESULTS

### Bacterial isolation and identification.

**FIG 1:**
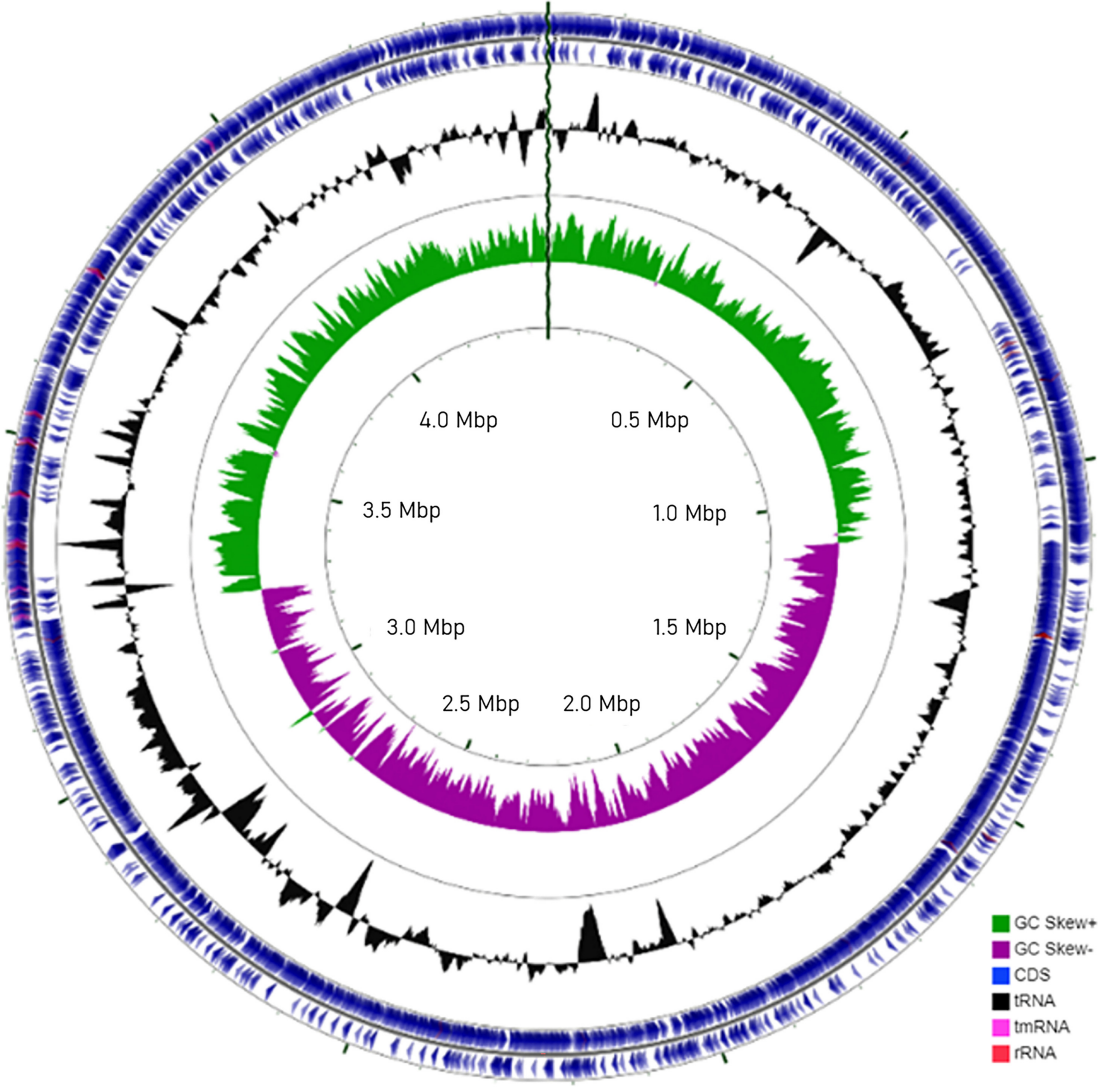
Circular map of the genome of the isolated *Alkalihalobacillus* sp. bacterium generated by the CGView comparison tool. Colored circles (from outside to inside) identify the (i) forward sequence feature, (ii) reverse sequence feature, (iii) GC content, and (iv) GC skew. Colors identify the genome contents as CDSs (blue), tRNAs (black), rRNAs (red), tmRNAs (pink), and positive (+) (green) or negative (−) (purple) GC skews.

**FIG 2:**
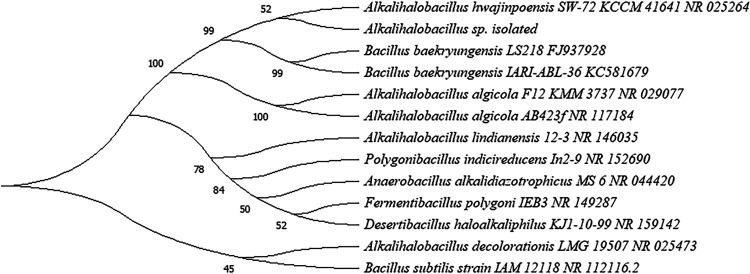
Phylogenetic closeness tree based on comparative analysis of the 16S rRNA gene sequences of the isolated *Alkalihalobacillus* sp. bacterium and phylogenetically closely related bacteria in the database. The evolutionary distances were computed using the maximum composite likelihood method. The tree was generated using the MEGAX tool according to the neighbor-joining method. A bootstrap test was performed in 1,000 replicates; the number next to each branch of the tree identifies the percentage of replicate trees in which the associated taxa were clustered together by the bootstrap test.

A single bacterial isolate was assigned for further research after it revealed polylyase activity in agar plates prepared with added *Ulva* polysaccharides and lacked this activity in control plates (see Fig. S1 in the supplemental material). Alignment of the intragenomic 16S rRNA gene copies revealed complete identity (100%) that confirmed the successful isolation of an axenic bacterium (Fig. S2). The whole-genome sequence was generated by combining data from the Illumina and Nanopore sequencing platforms. Nanopore sequencing generated 400 Mbp, of which 52,900 reads were of high quality (average length of 7,561 bp). The plasmid-free contig consisted of 4,461,509 bp with a mean coverage (average number of reads that align to known reference bases) of 87.48 and a GC content of 40.4%. The iterative polishing step against Illumina reads revealed nearly full identity of the aligned reads (99.89%), which were integrated into a single circular chromosome consisting of 4,463,966 bp with a mean coverage of 1195.9. The final resulting full genome consisted of 4,472 coding sequences (CDSs) annotated to 90 tRNA and 27 rRNA genes and a single transfer-messenger RNA (tmRNA) gene (Fig. 1). Phylogenetic analysis based on the whole genome proposed a novel bacterial strain with, and because of, low similarity to other available genomes. The most identical bacterium in the Microbial Genomes Atlas (MiGA) database was *Bacillus* sp. strain N1-1 (GenBank accession number CP046564), with an average nucleotide identity (ANI) score of 99.18% but a low coverage of only ~66%. In the JSpeciesWS database, identity was low, with a maximal ANI score of 77.05% or an overall genome-related index (OGRI) score of 0.97113, compared with Alkalihalobacillus macyae DSM 16346. Phylogenetic analysis based on the 16S rRNA gene revealed a high similarity of >99.5% in comparison with the bacterial strain Alkalihalobacillus hwajinpoensis SW-72 (Fig. 2).

### *In vitro* growth and α-amylase production.

**FIG 3:**
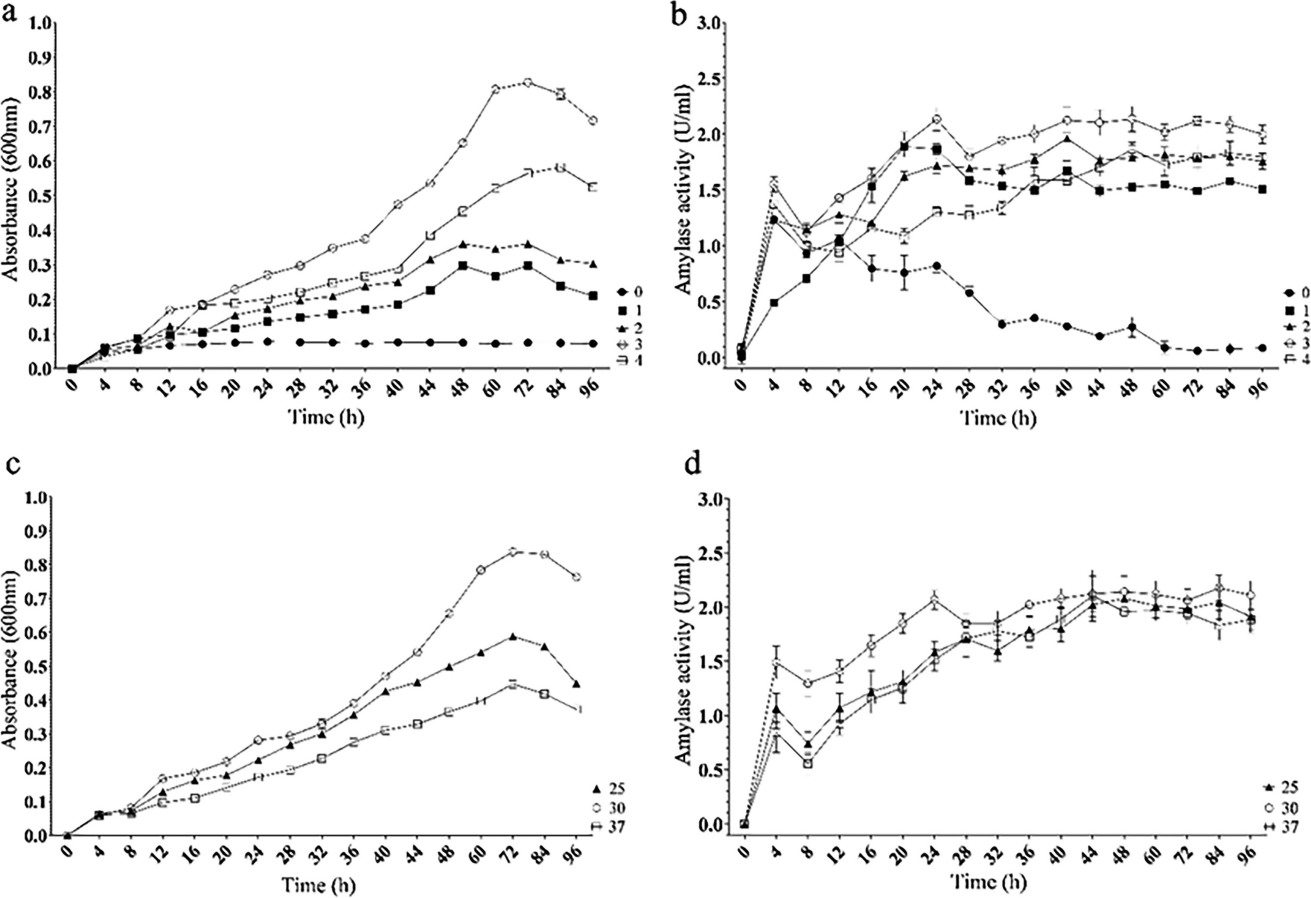
*In vitro* growth performance and α-amylase activity of the isolated *Alkalihalobacillus* sp. bacterium under different culture conditions. Bacterial growth (a and c) and α-amylase activity (b and d) were measured in cultures of different salinities (a and b) and temperatures (c and d) in a sampling regime of once every 4 h during the first 48 h and once every 12 h during the following 48-h period. One unit of α-amylase activity represents a rate of disappearance of 1 mg/min of the iodine binding starch in the assay reaction mixture. Values are means ± standard deviations (SD) (*n* = 3).

The growth of the isolated *Alkalihalobacillus* sp. was affected by salinity (*P* < 0.0001). Increasing the salinity up to the level of 3% was accompanied by higher bacterial yields and faster growth. Growth in salt-free cultures (0%) was nearly undetectable, and yields from 8 h post-inoculation remained similar (Fig. 3a; Table S1a). The highest yield was measured in cultures at 3% salinity after 72 h and was 1.4 to 2.8 times higher than the maximal yield in cultures at other salinities (Fig. 3b). The calculated generation time from fastest to slowest was 16, 20.3, 23.7, or 24 h in cultures at salinities of 3, 4, 2, or 1%, respectively.

The effect of temperature on bacterial growth was also significant (*P* < 0.0001), with the highest bacterial yields and fastest growth occurring at 30°C (Fig. 3c; Table S1c). At this temperature, the calculated bacterial generation time was 16.3 h, compared to 18.1 or 22 h at 25°C or 37°C, respectively.

The production of active α-amylases was affected by culture salinity (*P* < 0.0001), with the highest yields of α-amylases measured at 3% salinity (Fig. 3b; Table S1b). The most significant increase in the enzyme production yield was measured during the first 4 h of culture regardless of salinity. In cultures at 1, 2, or 3% salinity, enzyme production increased moderately until reaching maximal yields after 24 h, while at 4% salinity, the highest yield of α-amylases was measured 48 h after inoculation (Fig. 1). The yields of α-amylase in cultures incubated at different temperatures were relatively similar, with the highest yields at 30°C during the first 24 h of inoculation, after which relatively similar production yields were measured in the different cultures until the end of the experiment (Fig. 3d; Table S1d).

### Functional analysis of the genome of the *Alkalihalobacillus* sp.

Analysis of the whole-genome sequence of the *Alkalihalobacillus* sp. with the Rapid Annotations Using Subsystems Technology (RAST) server identified 4,598 CDSs. A total of 1,275 of these CDSs (28%) were successfully annotated into functional genes and clustered into 25 subsystem categories (Fig. S3). Many of the genes were clustered under the metabolism categories of amino acids and derivatives (372 genes), carbohydrates (274), and protein metabolism (236).

**FIG 4:**
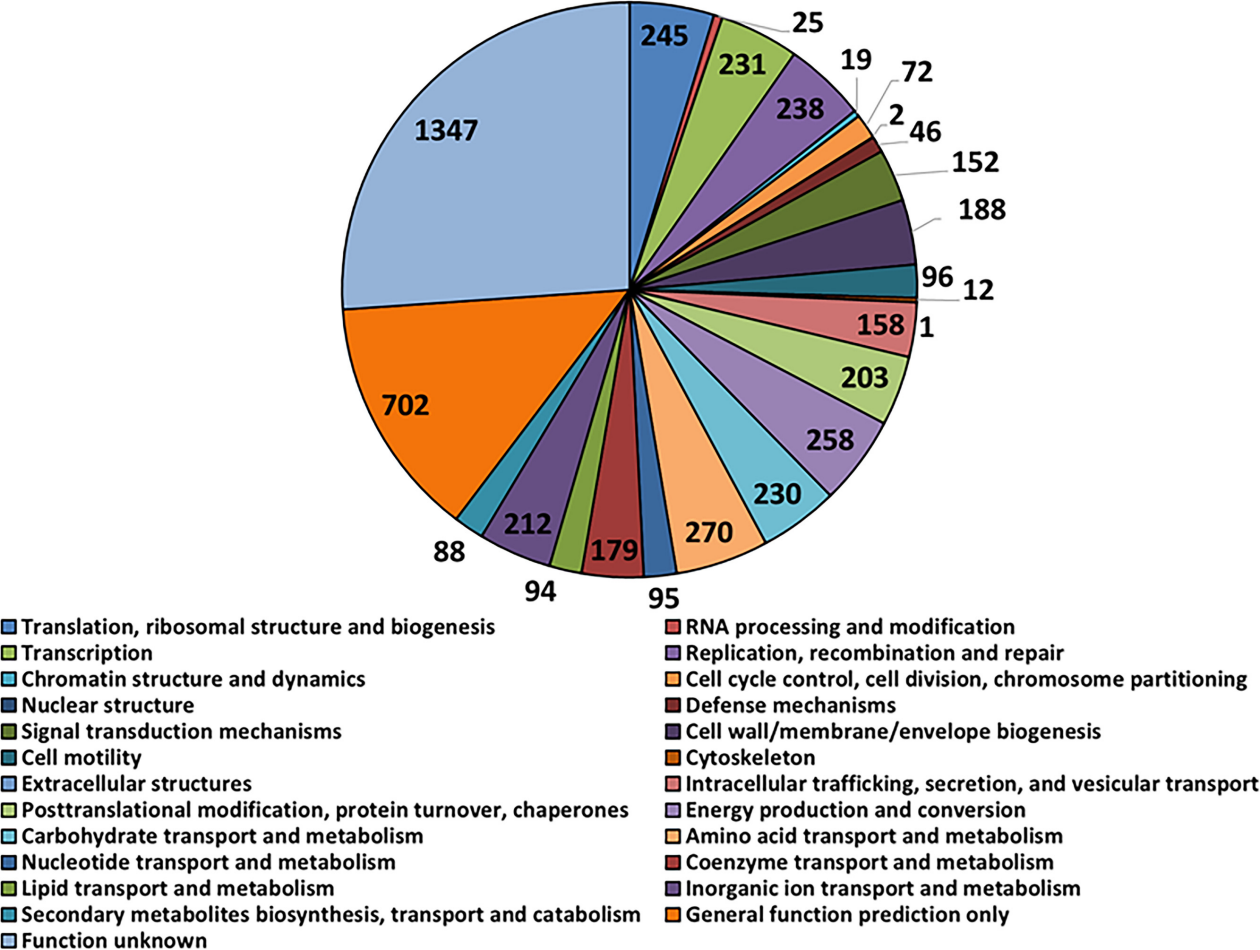
Pie chart of the contents of functional categories in the genome of the isolated *Alkalihalobacillus* sp. bacterium. Genes were annotated and categorized into Clusters of Orthologous Groups (COG), while the number of orthologue genes in each category is displayed.

Clusters of Orthologous Groups (COG) analysis identified 5,163 gene orthologues (GOs), 3,816 (74%) of which were successfully assigned to known protein-coding GOs and the rest of which were assigned to genes of either general or unknown function (Fig. 4). A total of 1,426 GOs were assigned to the category “metabolism,” most in the subcategories amino acid transport and metabolism (270 GOs), energy production (258), and conversion carbohydrate transport and metabolism (230). A total of 758 GOs were assigned to the category “information storage and processing,” most of which were in the subcategories translation (245 GOs); DNA replication, recombination, and repair (238); and transcription (231). The “cellular process and signaling” category was predominated by GOs of posttranslational modification, protein turnover, and chaperones (203 GOs) and cell wall/membrane/envelope biogenesis (188).

### The CAZome of *Alkalihalobacillus* sp.

The content of carbohydrate-active enzymes (CAZymes) in the genome is also referred as the bacterial CAZome. The *Alkalihalobacillus* sp. CAZome contained a pool of 94 copies of CAZymes with verified annotations (Fig. S4). CAZymes were clustered into 40 families: 19 GH families, 11 CBMs, 6 glycoside transferases (GTs), 3 carbohydrate esterases (CEs), and an additional single gene of auxiliary activities (AAs) (Table 1). Only CAZymes of the GT4 family reached a double-digit number of gene copies (16), followed by 9 gene copies each of the GT2, GH13, and GH32 CAZymes. The CBMs in the bacterial CAZome included modules for the binding of starch (CBM20, -34, and -41), cellulose, glycogen, peptidoglycan, chitin, inulin, levan, and xylan. GHs with an active site for these polysaccharides were also identified, with the highest number of subfamilies and gene copies for those targeting the α-glycoside/α-amylase linkage (GH13 and -5 subfamilies). No β-amylases were detected. Among the identified CEs, family CE4 had 6 gene copies for enzymes that catalyze the deacylation of polysaccharides.

**TABLE 1:** Various CAZyme genes in the genome of the isolated *Alkalihalobacillus* sp. bacterium^*a*^

Enzyme family	No. of gene copies	Function(s)
AA3_2	1	Glucose-methanol-choline oxidoreductases
CBM13	1	Cellulose-binding domain
CBM20	1	Starch binding
CBM34	2	Starch binding
CBM38	1	Inulin-binding function and hydrolyzation of fructose-containing polysaccharides
CBM4	1	Binding of xylan, β-1,3-glucan, β-1,3-1,4-glucan, β-1,6-glucan, and amorphous cellulose
CBM41	1	Starch binding
CBM48	4	Glycogen binding
CBM50	3	Binding of *N*-acetylglucosamine residues in bacterial peptidoglycans and chitin
CBM6	1	Binding of amorphous cellulose and β-1,4-xylan; some of these modules also bind β-1,3-glucan, β-1,3-1,4-glucan, and β-1,4-glucan
CBM66	1	Binding of fructose with higher affinity for inulin and levan
CBM68	1	Starch binding
CE14	2	*N*-Acetyl-1-d-myo-inosityl-2-amino-2-deoxy-α-d-glucopyranoside deacetylase
CE4	6	Catalyzes the deacylation of polysaccharides of acetyl-xylan, chitin, chitooligosaccharide, and peptidoglycan
CE9	1	Catalyzes the deacetylation of *N*-acetylglucosamine-6-phosphate to glucosamine-6-phosphate
GH1	3	β-Glucosidases and β-galactosidases
GH13	9	α-Amylases acting on substrates with α-glucoside linkages
GH15	2	Exo-acting enzymes that hydrolyze the nonreducing-end residues of α-glucosides
GH16_3	1	Active on β-1,4 or β-1,3 glycosidic bonds in glucans and galactans
GH18	2	Catalytically active chitinases
GH2	2	β-Galactosidases, β-glucuronidases, β-mannosidases, and exo-β-glucosaminidases
GH23	3	Lytic transglycosylases (also referred to as peptidoglycan lyases) of both bacterial and bacteriophage origins
GH3	1	Exo-acting β-d-glucosidases, α-l-arabinofuranosidases, β-d-xylopyranosidases, *N*-acetyl-β-d-glucosaminidases, and *N*-acetyl-β-d-glucosaminide phosphorylases
GH30_1	1	β-Glucosylceramidase, β-1,6-glucanase, and β-xylosidase
GH31	1	α-Glucosidases
GH32	9	Active on fructose-containing polysaccharides
GH35	1	β-Galactosidases
GH36	2	α-Galactosidase and α-*N*-acetylgalactosaminidase
GH38	1	Class II α-mannosidases
GH4	1	α-Glucosidases, α-galactosidases, α-glucuronidases, 6-phospho-α-glucosidases, and 6-phospho-β-glucosidases
GH42	1	β-Galactosidases
GH52	1	Exo-β-xylosidases
GH68	2	Levansucrase, β-fructofuranosidase, and inulosucrase
GH73	1	Cleaves the β-1,4-glycosidic linkage between *N*-acetylglucosaminyl and *N*-acetylmuramyl moieties in bacterial peptidoglycans
GT0	2	Catalyzes the transfer of sugar moieties from activated donor to acceptor molecules and forms glycosidic bonds
GT2	9	Transfers nucleotide diphosphate sugars to substrates such as polysaccharides and lipids
GT28	1	1,2-Diacylglycerol 3-β-galactosyltransferase, 1,2-diacylglycerol 3-β-glucosyltransferase, and β-*N*-acetylglucosamine transferase
GT4	16	Phospho-*N*-acetylmuramoyl-pentapeptide transferases
GT5	1	Involved in starch biosynthesis as part of glycan biosynthesis
GT51	5	Murein polymerase utilizing MurNAc-GlcNAc-P-P-lipid II as the sugar donor

a The different enzyme families in the genome are categorized as glycoside hydrolases (GHs), glycosyltransferases (GTs), carbohydrate esterases (CEs), carbohydrate-binding modules (CBMs), or auxiliary activity (AA). The number of gene copies of each enzyme family in the genome following genome analysis is provided together with the proposed functionality as identified in the CAZy database.

### Comparison of the *Alkalihalobacillus* sp. CAZome against those of other bacteria.

**FIG 5:**
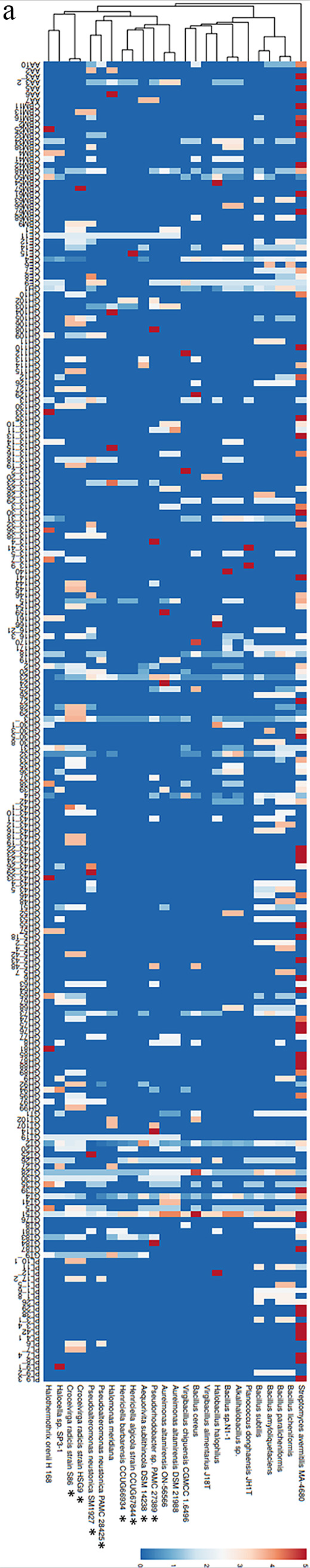

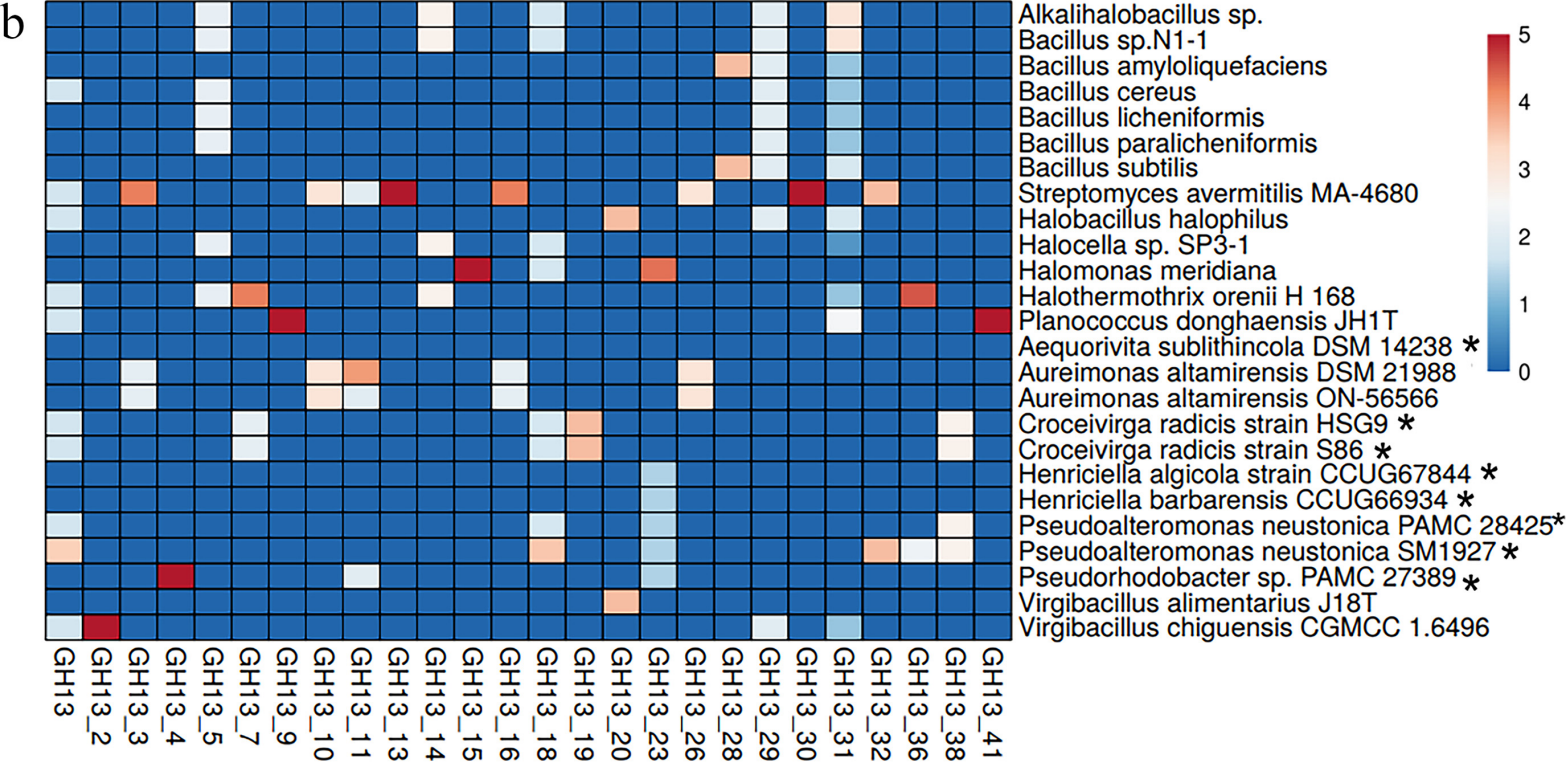
Comparative heat map diagrams of the entire contents of CAZymes (a) and GH13 family enzymes (b) in the genomes of the isolated *Alkalihalobacillus* sp. bacterium and other bacteria for which a complete genome is available in the database. The maps highlight the presence and the cumulative number of copies in the genome (dark blue [0] to dark red [5 or above]) of CAZymes of different families (a) or those of GH13 family only (b). A comparison of the isolated *Alkalihalobacillus* sp. was made against several species of *Bacillus* currently used for α-amylase production in various industries as well as against halophilic bacteria that contain hydrolases or polylyases in their genomes. Bacterial species that were reported for their capability of starch hydrolysis in *in vitro* experiments are marked with an asterisk. Similarity analysis based on the entire contents and number of copies of the CAZymes in the different bacteria was performed and is demonstrated by a similarity tree on the left side of the heat map in panel a.

A comparative analysis of the CAZomes of the isolated *Alkalihalobacillus* sp. and other selected bacteria (Table S2) revealed a total number of 224 CAZymes, with 140 GHs, 25 GTs, 21 polysaccharide lyases (PLs), 21 CBMs, 10 CEs, and 7 AAs (Fig. 5a). The CAZome of the halophilic bacterium Streptomyces avermitilis MA-4860 was the richest and contained 105 CAZymes, followed by two strains of the halophile Croceivirga radicis that consisted of 68 and 67 CAZymes. S. avermitilis MA-4860 also presented 38 unique CAZymes, many of them in numerous copies (>5). The poorest CAZome was from the bacterium Virgibacillus alimentarius J18T and contained only 11 CAZymes. Among the commercially important *Bacillus* species, B. paralicheniformis and B. cereus were the richest (66) and poorest (34) in CAZymes, respectively. Only the CAZyme families GT28 and GT51 were present in all examined bacteria, while GT2 and -4 were missing only in Pseudoalteromonas neustonica (SM1927). GH23 was also present in nearly all bacteria, excluding Halothermothrix orenii H168, while GH3 was not found in Bacillus cereus and Virgibacillus alimentarius J186.

The CAZome of the isolated *Alkalihalobacillus* sp. was most similar to that of the halophile *Bacillus* sp. N1-1 (Fig. 5a) and shared a pool of 36 CAZyme subfamilies. Twenty-one of these were GHs, 6 were CBMs, 5 were GTs, 3 were CEs, and 1 was an AA. Differences between these bacteria included a single GH4 gene in the CAZome of the *Alkalihalobacillus* sp. and 3 unique GHs of GH140, GH170, and GH171 in *Bacillus* sp. N1-1. The CAZyme families GH13 and GH43 were the richest in terms of their contents of genes from different subfamilies, with 26 and 15 subfamilies, respectively. Despite this richness in family GH43, 8 of the compared microbes lacked GH43 genes, while *B. paralicheniformis* and *S. avermitilis* each contained 8 CAZymes of this family. A closer examination of the α-amylases (GH13) revealed that only Aequorivita sublithincola DSM 14238 lacked CAZymes of this family and that Henriciella algicola CCUG67844, Henriciella barbarensis CCUG66934, and Virgibacillus alimentarius J18T had only a single copy of GH13 each. In contrast, *S. avermitilis* MA-4860 was also the richest in α-amylases, with 9 GH13 subfamilies, 2 of them unique to this bacterium. The most common GH13 CAZyme was GH13_31 of oligo-1,6-glucosidase, found in 12 of the examined bacteria. The isolated *Alkalihalobacillus* sp. and *Bacillus* sp. N1-1 were identical in their α-amylase contents, with each of these CAZymes appearing in identical numbers of copies (Fig. 5b). The GH13 CAZome of these two bacteria consisted of a relatively unique set of two α-amylases, GH13_14 and GH13_18, as both together were found only in *Halocella* sp. strain SP3-1.

### Starch metabolism by *Alkalihalobacillus* sp.

**FIG 6:**
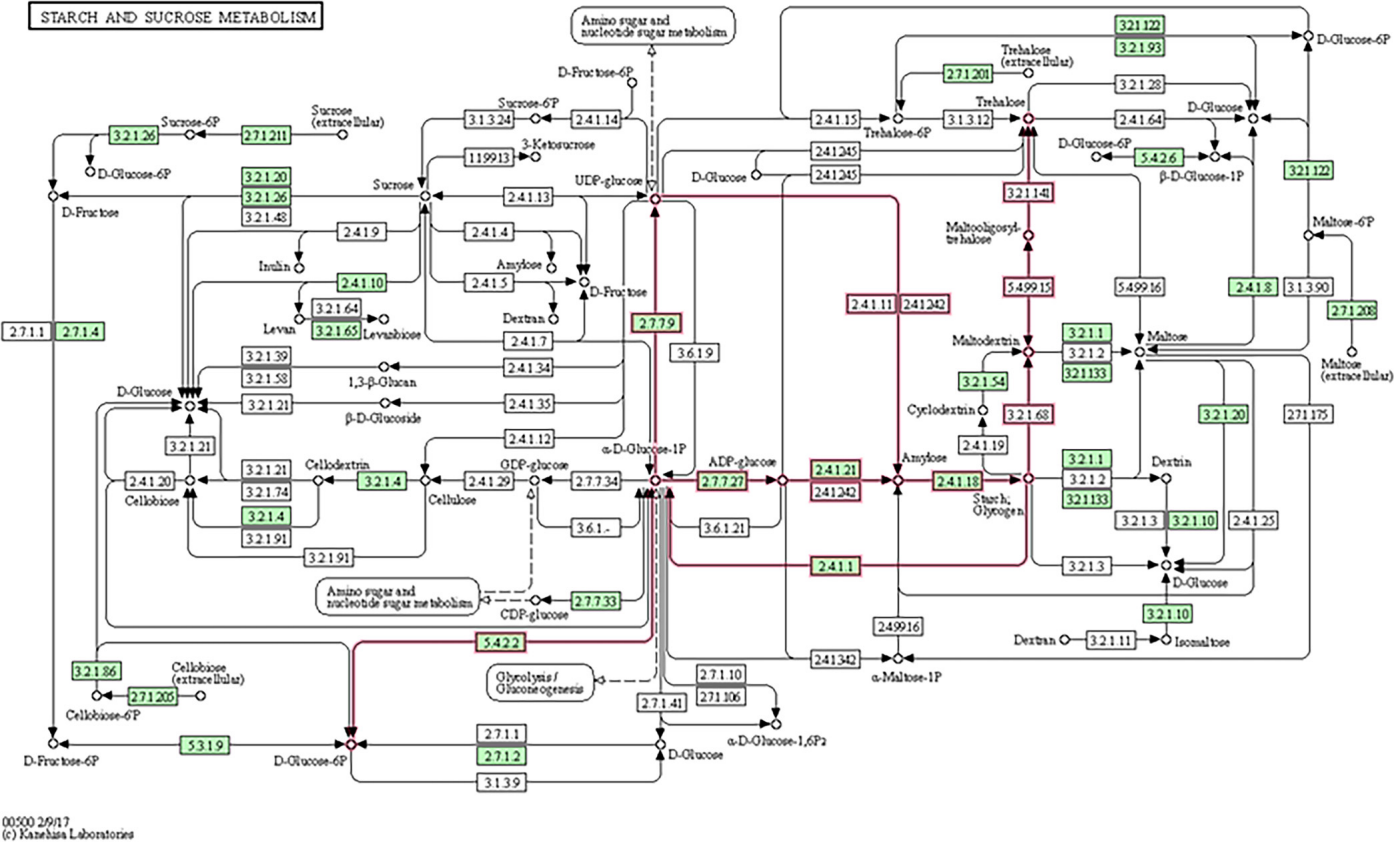
Hypothetical pathway for the metabolism of starch by the isolated *Alkalihalobacillus* sp. bacterium. Metabolic mapping was performed according to the Kyoto Encyclopedia of Genes and Genomes (KEGG). Green boxes represent the genes (CAZymes) that have been identified in the genome of the isolated bacterium, while clear boxes represent other known genes in the referred pathway between two given compounds (marked with ○). Arrows present the direction of the metabolic function. The highlighted red lines identify pathways for the complete metabolism of starch as suggested by KEGG Mapper.

The *Alkalihalobacillus* sp. CAZome revealed two catabolic pathways for the degradation of the starch substrate (Fig. 6). In the first pathway, glycogen phosphorylase (GH13_18; EC 2.4.1.1) breaks the linear chains in the glycogen substrate by phosphorolytic cleavages of the α-1,4-glucosidic bonds into α-d-glucose-1-phosphate oligo- or monosaccharides. In the following step, UTP-glucose-1-phosphate uridylyltransferase (GH13_29; EC 2.7.7.9) phosphorylates the α-d-glucose-1-phosphate to form another activated form of nucleotide glucose of a UDP-glucose, which is further utilized in metabolic cycles of amino or nucleotide sugars.

The second metabolic pathway involves the hydrolysis of starch into dextrin and maltose and their further conversion into d-glucose/dextrose units. The hydrolysis of starch is initiated by the attack on the α-1,4 glycoside bonds by α-amylase of either glucan (GH13_5; EC 3.2.1.1) or glucan 1,4-α-maltohydrolase (GH13_14; EC 3.2.1.133). In both cases, the hydrolysis of the starch substrate results in the formation of a dextrin polymer that is made up of d-glucose units and a maltose disaccharide made up of glucose units. The dextrin polymer is further hydrolyzed into d-glucose units by oligo-1,6-glucosidase (GH13_31; EC 3.2.1.10), which hydrolyzes the α-1,6 linkage in the polymer. In the alternative pathway through maltose, this substrate is hydrolyzed into d-glucose by maltose phosphorylase (GH31; EC 2.4.1.8).

## DISCUSSION

The current study revealed a new halophilic bacterial strain of an *Alkalihalobacillus* sp., whose genome contains a diverse pool of genes for carbohydrate-active enzymes. These include several α-amylases that enabled this bacterium to degrade starch in *in vitro* trials through the hypothetically complete metabolism of this polysaccharide into monosaccharides of d-glucose 6-phosphate (6P), d-glucose, and its active nucleotide form of UDP-glucose. Based on genomic analyses of both the whole-genome content of this bacterium and its 16S rRNA gene, we propose that this bacterium is a novel strain of the genus *Alkalihalobacillus*, referred to here as *Alkalihalobacillus* sp. Data from two comparative phylogenetic analyses of the whole-genome sequence were somewhat conflicting, as each allocated the isolated bacterium under a different taxonomic lineage, either *Alkalihalobacillus* or *Bacillus*. This misidentification may be attributed to the relatively low rate of coverage in the genome comparison to other bacteria, as only approximately 2/3 of the genome content of the bacterium isolated here aligned successfully against other bacteria in the database by either of the two genome comparison tools. The fact that the two comparisons differ in the BLAST algorithm that is used for the similarity analysis may have also contributed to the above-mentioned disagreement on bacterial identity (14). However, such uncertainty in the identity of this bacterium as either *Bacillus* or *Alkalihalobacillus* seems relatively common and therefore not very surprising. Recently, six species of the *Bacillus* genus were reclassified under the new genus taxon *Alkalihalobacillus* following a comparative genomic analysis (15). In fact, one of these reclassified microbes was Alkalihalobacillus hwajinpoensis SW-72, which was identified in the current study as that with the 16S rRNA gene sequence most similar to that of our bacterial isolate. Besides its genetic similarity, the A. hwajinpoensis SW-72 strain, isolated from the East China Sea in South Korea (16), also presents some physiological characteristics that are shared with our bacterial isolate, including its optimal growth at temperatures of 30°C to 35°C and 2 to 5% salinity, which is comparable to that of our isolate, which grew optimally at a temperature of 30°C and 3% salinity. However, while *A. hwajinpoensis* SW-72 was reported for its activity in the decomposition of various carbohydrates such as melibiose, d-cellobiose, d-ribose, d-galactose, d-mannose, or d-raffinose, no report was found on the potential activity of this strain in starch degradation, and its CAZome lacks genes for amylases (16). Moreover, a comparison of the genome of our isolated bacterium against a representative genome that is available in the database for the bacterial species *A. hwajinpoensis* (strain YJ1) did not result in sufficient similarity between the two bacteria, with only 54% of the genomes being aligned at a similarity level of just 82% (not shown). The similarity of the 16S rRNA sequence to those of members of the genus *Bacillus* supports the previous association of members of the *Alkalihalobacillus* genus with a distinct *Bacillus* clade (13).

This study’s *in vitro* trials confirmed that the isolated *Alkalihalobacillus* sp. bacterium is capable of growing and producing α-amylases at salinities of between 1 and 4% and temperatures of between 25°C and 37°C. However, both growth and α-amylase production were insignificant in the nonsaline cultures. Nevertheless, the stability of this bacterium’s α-amylases under this range of temperatures and salinities, and perhaps under more extreme conditions, has not been ruled out and is even likely. The thermostability of α-amylases was approved in several commercially used enzymes produced by *Bacillus* spp. (e.g., B. subtilis, B. licheniformis, or B. amyloliquefaciens), although these bacteria were all isolated from habitats of more moderate temperatures, such as the gastrointestinal tract of ruminants (10), soil (17), or plant roots (18). Additional evidence comes from a study of the marine bacterium Pseudoalteromonas neustonica SM1927, which was isolated from a 3% saline habitat and which revealed the optimal hydrolysis of starch at much higher salinities of between 6 and 10% in *in vitro* trials (19).

In the current study, we integrated the results from the *in vitro* experiments on the growth and production of α-amylases by the isolated bacterium with those from genomic analyses. This is to better understand the potential mechanism by which the starch substrate is metabolized and the relevant set of genes in such an activity. This is also crucial as in some cases, the efficient degradation of a substrate into the desired chemicals may require the construction of a set of enzymes that may not always be present in a single organism (e.g., bacterium). In this respect, the metabolism of starch in free-living bacteria has been proposed to require the following set of core genes (each in at least one copy): ADP-glucose pyrophosphorylase (GH13_18), glycogen synthase, glycogen phosphorylase, and branching and debranching enzymes. The *Alkalihalobacillus* sp. not only contains all of these in its genome but also was found to harbor several other genes related to starch metabolism from the families GH13_5, GH13_14, and GH13_31, which are involved in the metabolism of the maltooligosaccharides maltodextrin, maltose, and dextrin (3). The presence of GH13_31 and GH31 in the isolated *Alkalihalobacillus* sp. is highly important as these CAZymes are responsible for the consequent metabolism of the oligosaccharides dextrin and maltose into monosaccharides of d-glucose. If this process fails to occur, the entire metabolism of starch may be inhibited by the accumulation of either of these oligosaccharides (9). Due to this, we propose that the *Alkalihalobacillus* sp. isolated here allows the complete metabolism of starch into either d-glucose, d-glucose 6P, or UDP-glucose, with the latter as a precursor for amino sugar and nucleotide sugar metabolism.

The CAZome content of this isolated *Alkalihalobacillus* sp. reveals potential CAZymes for the hydrolysis of another commercially valuable polysaccharide, cellulose. The content of this polysaccharide in various seaweeds is relatively high. Among them are *Cladophora* spp. and *Ulva* spp. of the Chlorophyta, *Gracilaria* spp. and *Gelidium* spp. of the Rhodophyta, and Sargassum tennerimum and Saccharina japonica of the Ochrophyta (20). For this, we propose the hydrolases of *Alkalihalobacillus* sp. of β-glucosidases and β-galactosidases (both of the GH1 family) and a relevant cellulose-binding module of CBM13 or CBM66. The GH16 family of CAZymes in the *Alkalihalobacillus* sp. may be of great interest as these enzymes are active on the β-1,4 or β-1,3 glycoside bonds in various glucans and galactans (21). Together, the GH16 and CBM4 (or CBM6) CAZymes of the *Alkalihalobacillus* sp. may be efficient for the hydrolysis of laminarin, the primary polysaccharide in many brown seaweeds (e.g., *Saccharina* spp. and *Laminaria* spp.) (22).

Efficient processes to be developed with such enzymes may tip the balance to facilitate related processes for the extraction and purification of polysaccharides from seaweed and their hydrolysis in cost-effective biorefinery processes (23). Once available, such enzymes, if efficient, should facilitate the rapid expansion of seaweed aquaculture for commercial uses (6).

Our comparative analysis of bacterial CAZome contents encompassed specific members of the *Bacillus* genus that are currently used for the production of commercial α-amylases as well as several halophilic bacteria containing hydrolases or polylyases in their CAZomes. The results of this analysis agreed with those of whole-genome sequencing, as the highest similarity in each was recorded between the isolated *Alkalihalobacillus* sp. and the halophilic *Bacillus* sp. N1-1, which is also capable of starch degradation. However, the analyses also highlighted the fact that fundamental knowledge on bacterial CAZyme contents cannot be used as a sole indicator for enzymatic activity *per se*. The latter was evident in an examination of two strains of Croceivirga radicis that possess similar sets of five α-amylases in their genomes but were both found to be incapable of starch hydrolysis *in vitro* (24). In contrast, hydrolysis of starch has been reported in Virgibacillus alimentarius, although this bacterium contains only a single α-amylase of GH13_20 (in 4 copies) in its genome (25). We therefore propose that the data revealed in this study on the CAZome contents of selected bacteria will facilitate future *in vitro* trials toward the evaluation of their commercial potential.

The results of the current study suggest that this strain of *Alkalihalobacillus* sp. may be a novel source of α-amylases that may be beneficially applicable commercially in processes of starch degradation in temperate saline water of up to 4% salinity and perhaps higher. This can range from the treatment of starch-rich effluent from the textile or paper industries to biorefinery processes of starch-rich algae such as *Ulva* or *Gracilaria*. Besides α-amylases, the *Alkalihalobacillus* sp. contains other necessary enzymes for the hydrolysis of additional multibranch carbopolymers of glucose, glucans, or galactans found in other algal species like kelp. The comparative analyses of the phylogenetic closeness and CAZome content of the *Alkalihalobacillus* sp. suggest that this bacterium is a novel lineage of other starch-degrading bacteria of the genus *Bacillus* that specialize in saline habitats like the gut of sea urchins. The presence of CAZymes for the hydrolysis of the maltooligosaccharides maltose and dextrin in the *Alkalihalobacillus* sp. is highly important for the complete metabolism of starch into simple monosaccharides.

## MATERIALS AND METHODS

The workflow of the experimental work in the current research is demonstrated in a schematic diagram (see Fig. S1 in the supplemental material).

### Bacterial isolation.

The gut microbiome of the sea urchin Tripneustes gratilla, from an echinoculture system at the National Center for Mariculture in Eilat, Israel, was selected for the isolation of starch-hydrolyzing bacteria. This follows previous research that revealed that the gut of this sea urchin contains bacteria correlated with the starch-rich dietary seaweed *Ulva* or *Gracilaria* (26). To enrich the gut with such bacteria, adult sea urchins were fed a monospecific algal diet of Ulva fasciata for 8 weeks before harvesting the gut. Enrichment of gut bacteria continued in the laboratory in flasks with 100 mL of marine broth medium at 4% salinity (as in the Red Sea) with incubation at 25°C as in the echinoculture facility. This medium consists of the following (in grams per liter): peptone (5), yeast extract (1), C_6_H_5_FeO_7_ (0.1), NaCl (19.45), MgCl_2_ (5.9), MgSO_4_ (3.24), CaCl_2_ (1.8), KCl (0.55), NaHCO_3_ (0.16), KBr (0.08), and SrCl_2_ (0.034).

To enrich the targeted heterotrophic bacteria, we first reduced the contents of organic and inorganic residuals from the crude gut by diluting the culture medium by one-half every 3 days over 18 days using a similar bacterium-free medium. Three aliquots of 100 μL each were then sampled from each of the flasks and plated onto separate agar plates (1.5%) with the same medium. Bacterial colonies were transferred to new plates until their isolation. Each of the isolated colonies was transferred to a sterile flask containing 100 mL of mineral salts medium (4% salinity) and was also provided with a 2% polysaccharide extract from *Ulva* (extracted according to a method described previously by Trivedi et al. [27]) as the sole source of carbohydrates. After 24 h, 100 μL of the culture was plated onto agar plates with the same *Ulva* polysaccharide extract (2%) for 24 h at 37°C, followed by the examination of polylyase activity using a cetyl pyridinium chloride activity assay (28). A similar reaction was performed in triplicate as a control on agar plates without *Ulva* polysaccharide extract added. Only colonies that exhibited polylyase activity solely in the presence of *Ulva* polysaccharides but not in their absence were taken for analysis.

### *In vitro* growth and α-amylase activity measurements.

Bacterial growth and α-amylase activity were measured in cultures at different salinities and temperatures using the recommended liquid medium for starch-degrading bacteria (29) with 0.2% commercial soluble starch (Sigma-Aldrich, Israel).

In the first experiment, the isolated *Alkalihalobacillus* sp. was examined in cultures at different salinities of 0, 1, 2, 3, or 4% (Red Sea salinity). A subsequent experiment was performed at temperatures of 25°C, 30°C, and 37°C, all at a salinity level of 3%, which was identified in the first experiment as favorable for both growth and amylase activity. Both experiments included two sets of control treatments (in triplicates): the first was done using a starch-free culture medium with the isolated bacterium, and the second was done using a starch-containing, bacterium-free culture medium. In both experiments, 1 mL from an enriched culture of isolated *Alkalihalobacillus* bacteria was used as a starter. Bacterial growth was measured according to the cell density at 600 nm while sampling 1 mL from each culture once every 4 h during the first 48 h of the experiment and once every 12 h during the following 48 h. Measurement of the exocellular amylase activity was performed with a similar regime using the bacterium-free supernatant (100 μL) after centrifugation (10,000 rpm for 15 min). The amylase activity assay was the recommended starch-iodine assay in which the bacterium-free supernatant was added to 400 μL of a commercial 0.2% starch solution and incubated for 30 min at 50°C before stopping the activity with HCl and measuring the remaining starch with the iodine assay at 580 nm (30).

Statistical analysis of the data on growth or amylase activity was performed using two-way analysis of variance (ANOVA) to examine the effects of the various factors of time, salinity, and temperature.

### Genomic DNA extraction, sequencing, and annotation.

Bacterial genomic DNA was purified using the PureLink microbiome purification kit according to the manufacturer’s protocol. The quality and quantity of genomic DNA were determined using a Qubit fluorometer and an Agilent 2100 bioanalyzer. Whole-genome sequencing was performed using a MinION sequencer at the Research Resources Center (University of Illinois, Chicago, IL). The Porechop tool (v0.2.4) was used to trim adapters from the resulting sequence, while an additional removal of internal adapters was made at an identity threshold of 90% (31). Sequences shorter than 1,000 bp were discarded. *De novo* assembly was performed using the Canu assembler (32) with subsequent error correction using Racon (33). The Circlator platform (34) was used for the circularization of the contig assembly into a complete genome. The Illumina MiSeq platform and Nextera XT kit were used for the generation of short-read sequences, which were assigned together with the long-read sequence to an iterative polishing step for the removal of the remaining errors such as those in repeated regions. The Unicycler hybrid assembler (v0.4.1) integrated the data from the Illumina and Oxford Nanopore platforms to produce a complete assembly of a single chromosome (35). Finally, the sequences of contigs were corrected via multiple rounds of mapping read data to contigs by the Burrows-Wheeler Aligner v0.7.17 (36).

### Genome-based identification and taxonomic classification.

The 16S rRNA gene sequence was uploaded to the EzBioCloud server (37) to measure similarity against other sequences in the database according to ANI, while the All-Species Living Tree Project (38) and megablast (39) were used to align this gene with sequences in the SILVA and NCBI databases, respectively. The whole-genome sequence was aligned against those of other prokaryotic genomes in the NCBI database using MiGA (40), while overall genome-related index (OGRI) scores were calculated based on the tetranucleotide signatures from another alignment using the JSpeciesWS server (41) and data in GenomesDB.

### Genome functional annotation and comparative analysis of the CAZome contents.

Genome annotation was performed via the RAST server (42) and rapid Prokaryotic Genome Annotation software (Prokka) (43), followed by the generation of a genomic map in the CGView server (44). Orthologous genes were collected, functionally annotated, and clustered into categories using the Webmga (45) and BlastKOALA (46) servers and the COG (47) and Kyoto Encyclopedia of Genes and Genomes (KEGG) (48) databases, respectively. The genomic sequence was scanned for its content of CAZymes using DbCAN (49), with subsequent annotations into the enzyme classes GH, CBM, PL, GT, CE, or AA. Gene annotation was verified only when a similar annotation was identified in at least two of the three databases of HMMER, DIAMOND, and Hotpep. The DbCAN platform was used to compare the CAZome content of the isolated *Alkalihalobacillus* sp. against bacterial CAZomes in available genomes in the BacDive database (50), as listed in Table S1. Six were *Bacillus* species, known for their α-amylase production (including commercial ones) and capability of starch hydrolysis. In addition to *Bacillus*, the genomes of several halophilic bacteria were selected that either were reported to be capable of starch hydrolysis *in vitro* or presented a genome with either GH or PL CAZymes. Finally, a map of starch metabolism by the isolated *Alkalihalobacillus* sp. was constructed according to CAZyme content using KEGG Mapper (48).

### Data availability.

Nucleotide sequence data generated in this study are available in the European Nucleotide Archive with the project accession number PRJEB50008.

## References

[1] Tester RF, Karkalas J. 2005. Starch, p 423–480. *In* Steinbüchel A, Rhee SK (ed), Polysaccharides and polyamides in the food industry: properties, production, and patents. Wiley-VCH, Weinheim, Germany.

[2] Santana AL, Meireles MA. 2014. New starches are the trend for industry applications: a review. Food Public Health 4:229–241. doi:10.5923/j.fph.20140405.04.

[3] Stam MR, Danchin EGJ, Rancurel C, Coutinho PM, Henrissat B. 2006. Dividing the large glycoside hydrolase family 13 into subfamilies: towards improved functional annotations of α-amylase-related proteins. Protein Eng Des Sel 19:555–562. doi:10.1093/protein/gzl044. 17085431

[4] Jespersen HM, MacGregor EA, Sierks MR, Svensson B. 1991. Comparison of the domain-level organization of starch hydrolases and related enzymes. Biochem J 280:51–55. doi:10.1042/bj2800051. 1741756 PMC1130598

[5] Prabhu M, Chemodanov A, Gottlieb R, Kazir M, Nahor O, Gozin M, Israel A, Livney YD, Golberg A. 2019. Starch from the sea: the green macroalga Ulva ohnoi as a potential source for sustainable starch production in the marine biorefinery. Algal Res 37:215–227. doi:10.1016/j.algal.2018.11.007.

[6] Baghel RS, Trivedi N, Gupta V, Neori A, Reddy CRK, Lali A, Jha B. 2015. Biorefining of marine macroalgal biomass for production of biofuel and commodity chemicals. Green Chem 17:2436–2443. doi:10.1039/C4GC02532F.

[7] Crumling MA, King KA, Duncan RK. 2017. Cyclodextrins and iatrogenic hearing loss: new drugs with significant risk. Front Cell Neurosci 11:355. doi:10.3389/fncel.2017.00355. 29163061 PMC5676048

[8] Velázquez CF, Zamora LC, López GI, Meseguer OL, Núñez DE, Gabaldón JA. 2022. Cyclodextrins in polymer-based active food packaging: a fresh look at nontoxic, biodegradable, and sustainable technology trends. Polymers (Basel) 14:104. doi:10.3390/polym14010104. PMC8747138 35012127

[9] Saranraj DP, Naidu MA. 2013. Bacterial amylase: a review. Int J Pharm Biol Arch 4:274–287.

[10] Rabapane KJ, Mitema A, Nelson K, Feto NA. 2022. *Bacillus* spp. of ruminant origin as major sources of potential industrial amylases, p 209–230. *In* Islam MT, Rahman M, Pandey P (ed), Bacilli in agrobiotechnology: plant stress tolerance, bioremediation, and bioprospecting. Springer, Cham, Switzerland.

[11] Hwang CY, Lee I, Hwang YJ, Yoon SJ, Lee WS, Cho BC. 2016. *Pseudoalteromonas neustonica* sp. nov., isolated from the sea surface microlayer of the Ross Sea (Antarctica), and emended description of the genus *Pseudoalteromonas*. Int J Syst Evol Microbiol 66:3377–3382. doi:10.1099/ijsem.0.001202. 27260339

[12] Yamaguchi R, Tokunaga H, Ishibashi M, Arakawa T, Tokunaga M. 2011. Salt-dependent thermo-reversible α-amylase: cloning and characterization of halophilic α-amylase from moderately halophilic bacterium, *Kocuria varians*. Appl Microbiol Biotechnol 89:673–684. doi:10.1007/s00253-010-2882-y. 20871989

[13] Kumar S, Grewal J, Sadaf A, Hemamalini R, Khare S. 2016. Halophiles as a source of polyextremophilic α-amylase for industrial applications. AIMS Microbiol 2:1–26. doi:10.3934/microbiol.2016.1.1.

[14] Abouelhoda MI, Ohlebusch E. 2005. Chaining algorithms for multiple genome comparison. J Discrete Algorithms (Amst) 3:321–341. doi:10.1016/j.jda.2004.08.011.

[15] Patel S, Gupta RS. 2020. A phylogenomic and comparative genomic framework for resolving the polyphyly of the genus *Bacillus*: proposal for six new genera of *Bacillus* species, *Peribacillus* gen. nov., *Cytobacillus* gen. nov., *Mesobacillus* gen. nov., *Neobacillus* gen. nov., *Metabacillus* gen. nov. and *Alkalihalobacillus* gen. nov. Int J Syst Evol Microbiol 70:406–438. doi:10.1099/ijsem.0.003775. 31617837

[16] Yoon J-H, Kim I-G, Kang KH, Oh T-K, Park Y-H. 2004. *Bacillus hwajinpoensis* sp. nov. and an unnamed *Bacillus* genomospecies, novel members of *Bacillus* rRNA group 6 isolated from sea water of the East Sea and the Yellow Sea in Korea. Int J Syst Evol Microbiol 54:803–808. doi:10.1099/ijs.0.02678-0. 15143027

[17] Andualem B. 2014. Isolation and screening of amylase producing thermophilic spore forming bacilli from starch rich soil and characterization of their amylase activities using submerged fermentation. Int Food Res J 21:831–837.

[18] Fall R, Kinsinger RF, Wheeler KA. 2004. A simple method to isolate biofilm-forming Bacillus subtilis and related species from plant roots. Syst Appl Microbiol 27:372–379. doi:10.1078/0723-2020-00267. 15214643

[19] Jang GI, Lee I, Ha TT, Yoon SJ, Hwang YJ, Yi H, Yun S, Lee WS, Hwang CY. 2020. *Pseudomonas neustonica* sp. nov., isolated from the sea surface microlayer of the Ross Sea (Antarctica). Int J Syst Evol Microbiol 70:3832–3838. doi:10.1099/ijsem.0.004240. 32511084

[20] Baghel RS, Reddy CRK, Singh RP. 2021. Seaweed-based cellulose: applications, and future perspectives. Carbohydr Polym 267:118241. doi:10.1016/j.carbpol.2021.118241. 34119188

[21] Park BH, Karpinets TV, Syed MH, Leuze MR, Uberbacher EC. 2010. CAZymes Analysis Toolkit (CAT): Web service for searching and analyzing carbohydrate-active enzymes in a newly sequenced organism using CAZy database. Glycobiology 20:1574–1584. doi:10.1093/glycob/cwq106. 20696711

[22] Rioux LE, Turgeon SL. 2015. Seaweed carbohydrates, p 141–192. *In* Tiwari BK, Troy DJ (ed), Seaweed sustainability: food and non-food applications. Academic Press, San Diego, CA.

[23] Trincone A. 2018. Update on marine carbohydrate hydrolyzing enzymes: biotechnological applications. Molecules 23:901. doi:10.3390/molecules23040901. 29652849 PMC6017418

[24] Hu D, Wang L, Chen Y, Li X, Du Y, Sun F, Wang L, Fan C, Zhao R, Tang C, Shao Z. 2017. *Croceivirga radicis* gen. nov., sp. nov., isolated from a rotten tropical mangrove root. Int J Syst Evol Microbiol 67:3733–3738. doi:10.1099/ijsem.0.002179. 28895511

[25] Kim J, Jung M-J, Roh SW, Nam Y-D, Shin K-S, Bae J-W. 2011. *Virgibacillus alimentarius* sp. nov., isolated from a traditional Korean food. Int J Syst Evol Microbiol 61:2851–2855. doi:10.1099/ijs.0.028191-0. 21239563

[26] Masasa M, Kushmaro A, Kramarsky-Winter E, Shpigel M, Barkan R, Golberg A, Kribus A, Shashar N, Guttman L. 2021. Mono-specific algal diets shape microbial networking in the gut of the sea urchin *Tripneustes gratilla elatensis*. Anim Microbiome 3:79. doi:10.1186/s42523-021-00140-1. 34782025 PMC8594234

[27] Trivedi N, Baghel RS, Bothwell J, Gupta V, Reddy CRK, Lali AM, Jha B. 2016. An integrated process for the extraction of fuel and chemicals from marine macroalgal biomass. Sci Rep 6:30728. doi:10.1038/srep30728. 27470705 PMC4965815

[28] Ruijssenaars HJ, Hartmans S. 2001. Plate screening methods for the detection of polysaccharase-producing microorganisms. Appl Microbiol Biotechnol 55:143–149. doi:10.1007/s002530000477. 11330706

[29] Srivastava RAK, Baruah JN. 1986. Culture conditions for production of thermostable amylase by *Bacillus stearothermophilus*. Appl Environ Microbiol 52:179–184. doi:10.1128/aem.52.1.179-184.1986. 16347107 PMC203436

[30] Xiao Z, Storms R, Tsang A. 2006. A quantitative starch-iodine method for measuring alpha-amylase and glucoamylase activities. Anal Biochem 351:146–148. doi:10.1016/j.ab.2006.01.036. 16500607

[31] Wick RR, Judd LM, Gorrie CL, Holt KE. 2017. Completing bacterial genome assemblies with multiplex MinION sequencing. Microb Genom 3:e000132. doi:10.1099/mgen.0.000132. 29177090 PMC5695209

[32] Koren S, Walenz BP, Berlin K, Miller JR, Bergman NH, Phillippy AM. 2017. Canu: scalable and accurate long-read assembly via adaptive k-mer weighting and repeat separation. Genome Res 27:722–736. doi:10.1101/gr.215087.116. 28298431 PMC5411767

[33] Vaser R, Sović I, Nagarajan N, Šikić M. 2017. Fast and accurate de novo genome assembly from long uncorrected reads. Genome Res 27:737–746. doi:10.1101/gr.214270.116. 28100585 PMC5411768

[34] Hunt M, De Silva N, Otto TD, Parkhill J, Keane JA, Harris SR. 2015. Circlator: automated circularization of genome assemblies using long sequencing reads. Genome Biol 16:294. doi:10.1186/s13059-015-0849-0. 26714481 PMC4699355

[35] Wick RR, Judd LM, Gorrie CL, Holt KE. 2017. Unicycler: resolving bacterial genome assemblies from short and long sequencing reads. PLoS Comput Biol 13:e1005595. doi:10.1371/journal.pcbi.1005595. 28594827 PMC5481147

[36] Li H. 2013. Aligning sequence reads, clone sequences and assembly contigs with BWA-MEM. arXiv 10.48550/arXiv.1303.3997.

[37] Yoon SH, Ha SM, Kwon S, Lim J, Kim Y, Seo H, Chun J. 2017. Introducing EzBioCloud: a taxonomically united database of 16S rRNA gene sequences and whole-genome assemblies. Int J Syst Evol Microbiol 67:1613–1617. doi:10.1099/ijsem.0.001755. 28005526 PMC5563544

[38] Yilmaz P, Parfrey LW, Yarza P, Gerken J, Pruesse E, Quast C, Schweer T, Peplies J, Ludwig W, Glöckner FO. 2014. The SILVA and “All-Species Living Tree Project (LTP)” taxonomic frameworks. Nucleic Acids Res 42:D643–D648. doi:10.1093/nar/gkt1209. 24293649 PMC3965112

[39] Johnson M, Zaretskaya I, Raytselis Y, Merezhuk Y, McGinnis S, Madden TL. 2008. NCBI BLAST: a better Web interface. Nucleic Acids Res 36:W5–W9. doi:10.1093/nar/gkn201. 18440982 PMC2447716

[40] Rodriguez-R LM, Gunturu S, Harvey WT, Rosselló-Mora R, Tiedje JM, Cole JR, Konstantinidis KT. 2018. The Microbial Genomes Atlas (MiGA) webserver: taxonomic and gene diversity analysis of Archaea and Bacteria at the whole genome level. Nucleic Acids Res 46:W282–W288. doi:10.1093/nar/gky467. 29905870 PMC6031002

[41] Richter M, Rosselló-Móra R, Glöckner FO, Peplies J. 2016. JSpeciesWS: a Web server for prokaryotic species circumscription based on pairwise genome comparison. Bioinformatics 32:929–931. doi:10.1093/bioinformatics/btv681. 26576653 PMC5939971

[42] Aziz RK, Bartels D, Best A, DeJongh M, Disz T, Edwards RA, Formsma K, Gerdes S, Glass EM, Kubal M, Meyer F, Olsen GJ, Olson R, Osterman AL, Overbeek RA, McNeil LK, Paarmann D, Paczian T, Parrello B, Pusch GD, Reich C, Stevens R, Vassieva O, Vonstein V, Wilke A, Zagnitko O. 2008. The RAST server: rapid annotations using subsystems technology. BMC Genomics 9:75. doi:10.1186/1471-2164-9-75. 18261238 PMC2265698

[43] Seemann T. 2014. Prokka: rapid prokaryotic genome annotation. Bioinformatics 30:2068–2069. doi:10.1093/bioinformatics/btu153. 24642063

[44] Grant JR, Stothard P. 2008. The CGView server: a comparative genomics tool for circular genomes. Nucleic Acids Res 36:W181–W184. doi:10.1093/nar/gkn179. 18411202 PMC2447734

[45] Wu S, Zhu Z, Fu L, Niu B, Li W. 2011. WebMGA: a customizable Web server for fast metagenomic sequence analysis. BMC Genomics 12:444. doi:10.1186/1471-2164-12-444. 21899761 PMC3180703

[46] Kanehisa M, Sato Y, Morishima K. 2016. BlastKOALA and GhostKOALA: KEGG tools for functional characterization of genome and metagenome sequences. J Mol Biol 428:726–731. doi:10.1016/j.jmb.2015.11.006. 26585406

[47] Tatusov RL, Galperin MY, Natale DA, Koonin EV. 2000. The COG database: a tool for genome-scale analysis of protein functions and evolution. Nucleic Acids Res 28:33–36. doi:10.1093/nar/28.1.33. 10592175 PMC102395

[48] Kanehisa M, Goto S. 2000. KEGG: Kyoto Encyclopedia of Genes and Genomes. Nucleic Acids Res 28:27–30. doi:10.1093/nar/28.1.27. 10592173 PMC102409

[49] Yin Y, Mao X, Yang J, Chen X, Mao F, Xu Y. 2012. DbCAN: a Web resource for automated carbohydrate-active enzyme annotation. Nucleic Acids Res 40:W445–W451. doi:10.1093/nar/gks479. 22645317 PMC3394287

[50] Reimer LC, Vetcininova A, Carbasse JS, Söhngen C, Gleim D, Ebeling C, Overmann J. 2019. Bac*Dive* in 2019: bacterial phenotypic data for high-throughput biodiversity analysis. Nucleic Acids Res 47:D631–D636. doi:10.1093/nar/gky879. 30256983 PMC6323973

