# Supplementary material for: Carbohydrate-Active Enzymes of a Novel Halotolerant Alkalihalobacillus Species for Hydrolysis of Starch and Other Algal Polysaccharides
Source: Microbiol Spectr. 2022 Jul 11;10(4):e01078-22. doi: 10.1128/spectrum.01078-22 (PMC9431708; doi:10.1128/spectrum.01078-22)
Supplement: Supplemental file 1 — Supplemental material. Download spectrum.01078-22-s0001.pdf, PDF file, 0.6 MB [file spectrum.01078-22-s0001.pdf]

Supplementary material

Figures

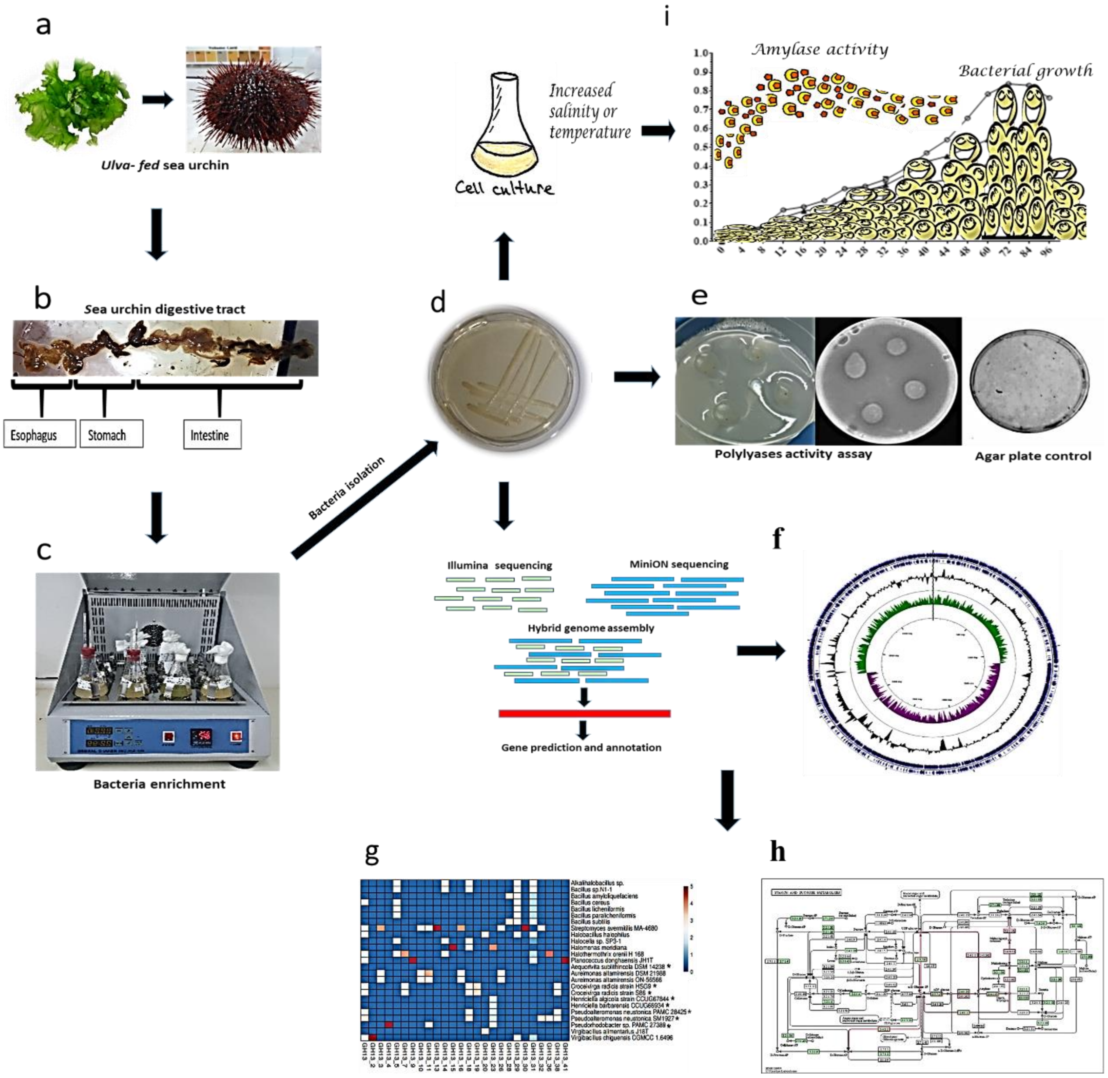

FIG S1 A graphical overview of the workflow in the current research including the following steps: a. feeding sea urchins a mono-specific algal diet of *Ulva fasciata* for 8 weeks; b. harvesting the whole digestive tract of the animals; c. enrichment of the heterotrophic bacterial community in the crude gut samples through incubation in marine broth media; d. Isolation of bacterial colonies on agar plates with marine broth media; e. polylyases activity assay on isolated bacterial colonies performed on agar plates with mineral salts media and *Ulva*-polysaccharides extract, revealing one bacterium that had such functionality in the presence of *Ulva*-polysaccharides and none

without them; f. whole genome sequencing of the bacterial isolate and its identification as a new species of *Alkalihalobacillus sp.*, examined through similarity measurement using ANI, and OGRI identity scores and similarity in the 16S rRNA gene against other bacteria in the database; g. analysis of the CAZome content in *Alkalihalobacillus sp.* and its comparison to other selected *Bacillus* bacteria with commercially used amylases, or halophiles that are either capable of *in vitro* starch hydrolysis or carry GHs or PLs in their CAZome; h. KEGG mapping of starch metabolism by *Alkalihalobacillus sp.;* i. *in vitro* trials measured bacterial growth and α-amylase activity in cultures with *Alkalihalobacillus sp.* at various salinities or temperatures.

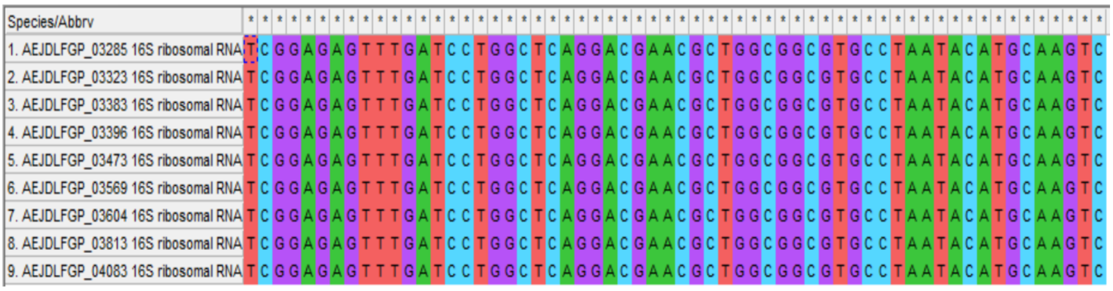

FIG S2 Alignment of the intragenomic gene copies in the genome of isolated *Alkalihalobacillus sp*. The nine 16S rRNA gene copies were extracted from the whole genome sequencing data in order to verify the axenicity of the isolated bacterium using the similarity threshold of >98.65.

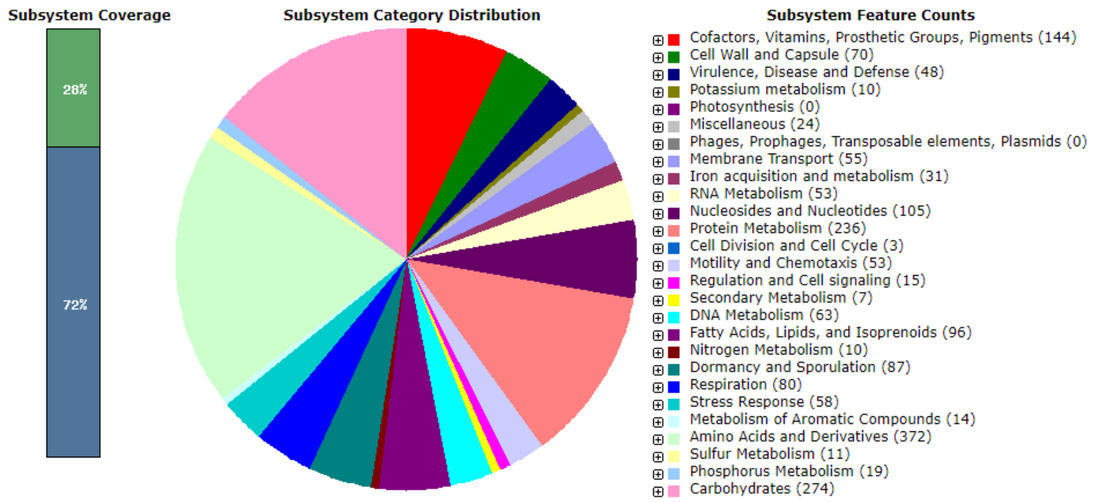

FIG S3 A pie chart of the functional categories (as subsystems) in the genome of *Alkalihalobacillus sp.* as analyzed in RAST. Colors indicates the subsystem category as per the index on the right while the number of annotated genes in each category is indicated in brackets. The left column bar presents the coverage of gene analysis with the percent of genes that were either clustered (green) or not clustered (blue) into known subsystem categories.

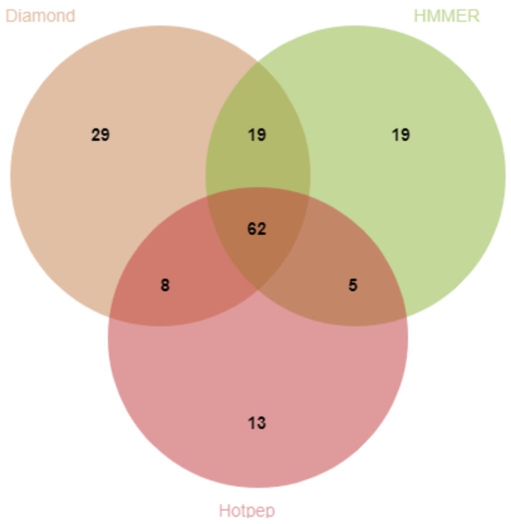

FIG S4 A Venn diagram presents the results on the annotated genes of *Alkalihalobacillus sp.* using the three different tools of HMMER (green), DIAMOND (brown), or Hotpep (pink). Only genes that received a similar annotation in at least two of the three tools were considered as 'truly annotated'.

Tables

TABLE S1 Statistical analyses of the two-way ANOVA test for the *in vitro* trial with *Alkalihalobacillus sp.* examining the effects of different salinities (a,b), or temperatures (c,d), as well as of culture time (a,b,c,d), on bacterial growth (a,c) or amylase activity (b,d). The impact of integrated factors of culture time and salinity or culture time and temperature was also examined and presented under the term ‘interaction’.

a	Optimum salinity for bacterial growth					
Source of Variation	% of total variation	P value	SS	DF	MS	F (DFn, DFd)
Interaction (time+salinity)	21.78	<0.0001	2.1	64	0.03281	F (64, 170) = 942.1
Time	44.6	<0.0001	4.299	16	0.2687	F (16, 170) = 7716
Salinity	33.55	<0.0001	3.234	4	0.8086	F (4, 170) = 23219
Residual			0.00592	170	3.482E-05	
b	Optimum salinity for amylase activity					
Source of Variation	% of total variation	P value	SS	DF	MS	F (DFn, DFd)
Interaction (time+salinity)	20.83	<0.0001	21.92	64	0.3425	F (64, 170) = 30.18
Time	29.55	<0.0001	31.09	16	1.943	F (16, 170) = 171.2
Salinity	47.78	<0.0001	50.27	4	12.57	F (4, 170) = 1107
Residual			1.929	170	0.01135	
c	Optimum temperature for bacterial growth					
Source of Variation	% of total variation	P value	SS	DF	MS	F (DFn, DFd)
Interaction (time+temperature)	7.779	<0.0001	0.5691	32	0.01778	F (32, 102) = 641.0
Time	81.91	<0.0001	5.992	16	0.3745	F (16, 102) = 13499
Temperature	10.28	<0.0001	0.7518	2	0.3759	F (2, 102) = 13550
Residual			0.00283	102	2.774E-05	
d	Optimum temperature for amylase activity					
Source of Variation	% of total variation	P value	SS	DF	MS	F (DFn, DFd)
Interaction (time+temperature)	3.09	<0.0001	1.556	32	0.04862	F (32, 102) = 3.348
Time	88.41	<0.0001	44.52	16	2.782	F (16, 102) = 191.6
Temperature	5.556	<0.0001	2.797	2	1.399	F (2, 102) = 96.32
Residual			1.481	102	0.01452	

TABLE S2 A list of the selected bacteria with representative genomes in the NCBI database (including Bio-project number) that were used for CAZyme comparison. The

table also indicates if the bacterial species are reported capable of starch degradation (including reference) and whether their CAZomes contain GHs or PLs. Halophile species are also indicated.

**Species:**	**Bio-project**	***In vitro* starch hydrolysis (with reference)**	**Halophile**	
Croceivirga radicis HSG9	PRJNA358143	-	+	Hu et al., 2017
Croceivirga radicis S86	PRJNA65289	-	+	Hu et al., 2017
Pseudoalteromonas neustonica PAMC 28425	PRJDB4787	-	+	Hwang et al., 2016
Pseudoalteromonas neustonica SM1927	PRJNA554250	-	+	Hwang et al., 2016
Pseudorhodobacter PAMC27389	PRJNA286976	-	+	Lee et al., 2016
Aureimonas altamirensis DSM21988	PRJEB18307	+	+	Jurado et al. 2006
Aureimonas altamirensis ON-56566	PRJNA260421	+	+	Jurado et al. 2006
Aequorivita sublithincola DSM14238	PRJNA46631	-	+	Lucas et al. 2012
Henriciella algicola CCUG67844	PRJNA381091	-	+	Rathsack et al. 2006
Henriciella barbarensis CCUG66934	PRJNA381091	-	+	Rathsack et al. 2006
Virgibacillus alimentarius J18T	PRJNA238855	+	+	Kim et al. 2011
Virgibacillus chiguensis CGMCC1.6496	PRJEB18238	+	+	Wang et al. 2008
Bacillus subtilis	PRJNA76	+	-	Konsula et al. 2004
Bacillus cereus	PRJNA719349	+	-	Annamalai et al. 2011
Bacillus licheniformis	PRJNA293170	+	-	Saito et al. 1973
Bacillus amyloliquefaciens	PRJNA73591	+	-	Deb et al. 2013
Bacillus paralicheniformis	PRJNA412146	+	-	Božić et al. 2020
Halomonas meridiana	PRJNA416452	+	+	Coronado et al. 2000
Halocella sp. SP3-1	PRJNA493542	+	+	Heng et al. 2019
Planococcus donghaensis JH1T	PRJNA60823	+	+	Too et al. 2017
Halothermothrix orenii H168	PRJNA16377	+	+	Tan et al. 2008
Streptomyces avermitilis MA-4680	PRJNA189	+	+	Samiea et al. 2012
Halobacillus halophilus	PRJEA50647	+	+	Amoozegar et al. 2003
Bacillus sp. N1-1	PRJNA592026	+	+	Wang et al. 2020
Alkalihalobacillus sp.		+	+	**Current study**
